# A Systematic Review of Cardiovascular Health Screening in Michigan: Are We Doing Enough?

**DOI:** 10.7759/cureus.100333

**Published:** 2025-12-29

**Authors:** Shadan Alrawi, Andreanna C Ulery, Manasvi Patel, Shafi Mohamed

**Affiliations:** 1 College of Literature, Science, and the Arts, University of Michigan, Ann Arbor, USA; 2 Cardiothoracic Surgery, Memorial Healthcare, Lufkin, USA

**Keywords:** access to healthcare, awareness of cardiovascular diseases, cardio vascular disease, cardiovascular prevention, preventive screening, rural michigan

## Abstract

Cardiovascular disease (CVD) remains the leading cause of death in the United States, with persistent disparities in access to preventive screening. This systematic review investigates how racial, geographic, and socioeconomic factors influence cardiovascular screening rates across Michigan, focusing on blood pressure and cholesterol screening across various groups. A systematic search of PubMed, Embase, Scopus, Web of Science, and Google Scholar was conducted for studies published through October 2025. Eligible studies reported Michigan-specific data on preventive cardiovascular screening, operationally defined as blood pressure measurement, and/or lipid testing in adults. Twelve studies met the inclusion criteria, and methodological quality was assessed using study-design-appropriate appraisal tools, including the NIH quality assessment instrument. Across studies, Black, rural, uninsured, and low-income populations consistently demonstrated lower screening rates compared with White, urban, and insured groups. Reported disparities in screening ranged from approximately 5%-15% points across racial and socioeconomic strata, depending on population and data source. Policy and community-based interventions, including Medicaid expansion and mobile screening initiatives, were associated with modest increases in screening, although effects were variable and not uniformly sustained. Overall, preventive cardiovascular screening in Michigan remains unevenly distributed, highlighting persistent equity gaps and the need for more consistent, statewide screening strategies.

## Introduction and background

Cardiovascular disease (CVD) remains the leading cause of mortality in the United States. In 2023, roughly 919,000 Americans died of CVD (about one in three deaths) [1]. Heart disease alone kills one person every ~34 seconds and costs the nation hundreds of billions of dollars, annually [1]. Key modifiable CVD risk factors, notably high blood pressure (hypertension) and high cholesterol, are widespread in the population [1]. Consequently, national guidelines strongly emphasize preventive screening, including routine blood pressure and lipid checks in adults, to identify and treat these risk factors early [1]. Early detection through screening has been shown to reduce CVD events, making it an important step in CVD prevention in public health practice [1,2]. In this review, *preventive cardiovascular screening* refers specifically to routine measurement of blood pressure and testing for blood cholesterol levels in adults, consistent with national preventive care guidelines.

Michigan bears a heavy cardiovascular burden. The Michigan Department of Health and Human Services (MDHHS) reports that heart disease has been the state’s leading cause of death for more than a decade [1]. In 2019, 25,500 Michigan residents died of heart disease, and another 5,159 died of stroke [1]. Although mortality rates have fallen modestly in recent years, they remain higher than national averages (Michigan’s age-adjusted heart disease death rate declined only 5% from 2009 to 2019 vs. 10% nationally) [1,3]. Large disparities underlie these averages: for example, MDHHS data show non-Hispanic Black Michigan residents have the highest heart disease mortality rate of any racial/ethnic group in the state [3]. Controllable risk factors such as tobacco use, obesity, hypertension, and dyslipidemia are common [1,4]. According to MDHHS and a national survey, roughly one-third of U.S. adults have multiple CVD risk factors, and only about 36% have none [1,4]. In Michigan, the prevalence of hypertension and high cholesterol is roughly comparable to, or slightly higher than, national levels (e.g., ~36% of Michigan adults report hypertension vs. ~34% nationally) [3,5]. Thus, regular screening for blood pressure and cholesterol is critical, yet Michigan’s preventive care utilization shows gaps by subgroup [4]. While these statistics describe downstream cardiovascular outcomes, the present review focuses on upstream disparities in preventive screening that precede disease detection and management.

In Michigan, disparities in access to preventive CVD screening have been documented across geography, race/ethnicity, and socioeconomic status [3,4].

Geography

Rural residents face higher CVD risk and poorer access to care. Rural Michigan counties report self-reported hypertension in 41%-55% of adults, far above the ~35% statewide average [3,4,6]. Rural areas also have fewer primary care providers, leading to lower rates of routine checkups and poorer blood pressure control [3,6].

Race/ethnicity

African American Michigan residents experience the worst outcomes and have the highest heart disease and stroke death rates in the state [3]. MiBRFSS data show that Black adults with hypertension are more likely than whites to have had a stroke, indicating both a higher burden of disease and gaps in effective screening and management [3,4]. Screening data also hint at disparities; for example, Hispanic individuals in Michigan report lower rates of cholesterol testing (86.5% ever tested vs. 92% among non-Hispanic White individuals) [4].

Socioeconomic status

Lower income and education are linked to less preventive care [3,4]. Nearly 18%-21% of Michigan adults in the lower income or education brackets reported no routine doctor visit in the past year, reducing opportunities for blood pressure or cholesterol checks [4]. Correspondingly, recent MiBRFSS tables show adults with <$20,000 incomes are less likely to have had a recent cholesterol test than those with higher incomes (85.8% vs. 91.2% in the past five years) [4]. Men, younger adults, and uninsured people are also more likely to miss preventive visits, compounding these gaps [4]. These surveillance datasets are informative not only of cardiovascular risk burden but also of differential utilization of preventive screening services across population subgroups in Michigan. In sum, Michigan residents who live in rural areas, are racial/ethnic minorities, or have lower socioeconomic status tend to have lower screening rates and higher CVD risk.

Disparities in screening and outcomes can be summarized as follows:

Geography (Rural vs. Urban): Rural Michigan suffers disproportionately high hypertension prevalence and CVD mortality, compounded by primary care shortages [3,6]. Preventive screening rates are lower in rural areas, likely due to access barriers.

Race/Ethnicity: Non-Hispanic Black residents of Michigan have markedly higher CVD mortality and are less likely to receive preventive services than White residents, contributing to worse outcomes [3,7]. Hispanic and other racial or ethnic minority groups similarly show lower screening and control rates than non-Hispanic White residents.

Socioeconomic status: Lower-income/education groups are less likely to have routine checkups or screening. For instance, only ~86% of very-low-income Michigan residents had their cholesterol checked in the past five years, compared with ~91% of those with higher incomes; nearly one-quarter of adults with lower educational attainment skipped annual exams. These care gaps align with the inverse equity pattern of CVD risk factors and outcomes [4,8].

Several state and local initiatives have sought to improve preventive screening in Michigan. The Healthy Hearts for Michigan (HH4M) program, an Agency for Healthcare Research and Quality (AHRQ)-sponsored quality improvement cooperative, works with rural primary care practices to improve hypertension management and smoking cessation [6]. Similarly, Michigan has piloted community-based pharmacy screening programs (e.g., pharmacy blood pressure check stations) and mobile clinics to reach underserved populations [3,9]. These interventions have shown promise in boosting screening in targeted settings, but their scope remains limited [6,9]. Most programs cover a subset of communities or rely on external funding, and none have yet eliminated the underlying disparities across the state [3,10]. In particular, these efforts tend to address a single risk factor or population (e.g., hypertension in rural clinics) rather than comprehensively addressing screening disparities for all major CVD risk factors statewide [3,6].

The objective of this systematic review is to synthesize Michigan-specific evidence on disparities in preventive cardiovascular screening across racial, geographic, and socioeconomic groups. To our knowledge, there has been no systematic review focused on preventive cardiovascular screening disparities specific to Michigan. Existing publications are largely single-state analyses (e.g., MiBRFSS reports and local studies) or national reviews that do not disaggregate Michigan’s context [4,7,11]. Thus, a systematic synthesis of the Michigan-specific evidence on who is being screened and who is being missed for hypertension and dyslipidemia is lacking. This review aims to fill that gap by summarizing the literature on preventive CVD screening disparities in Michigan.

## Review

Methodology

**Figure 1:**
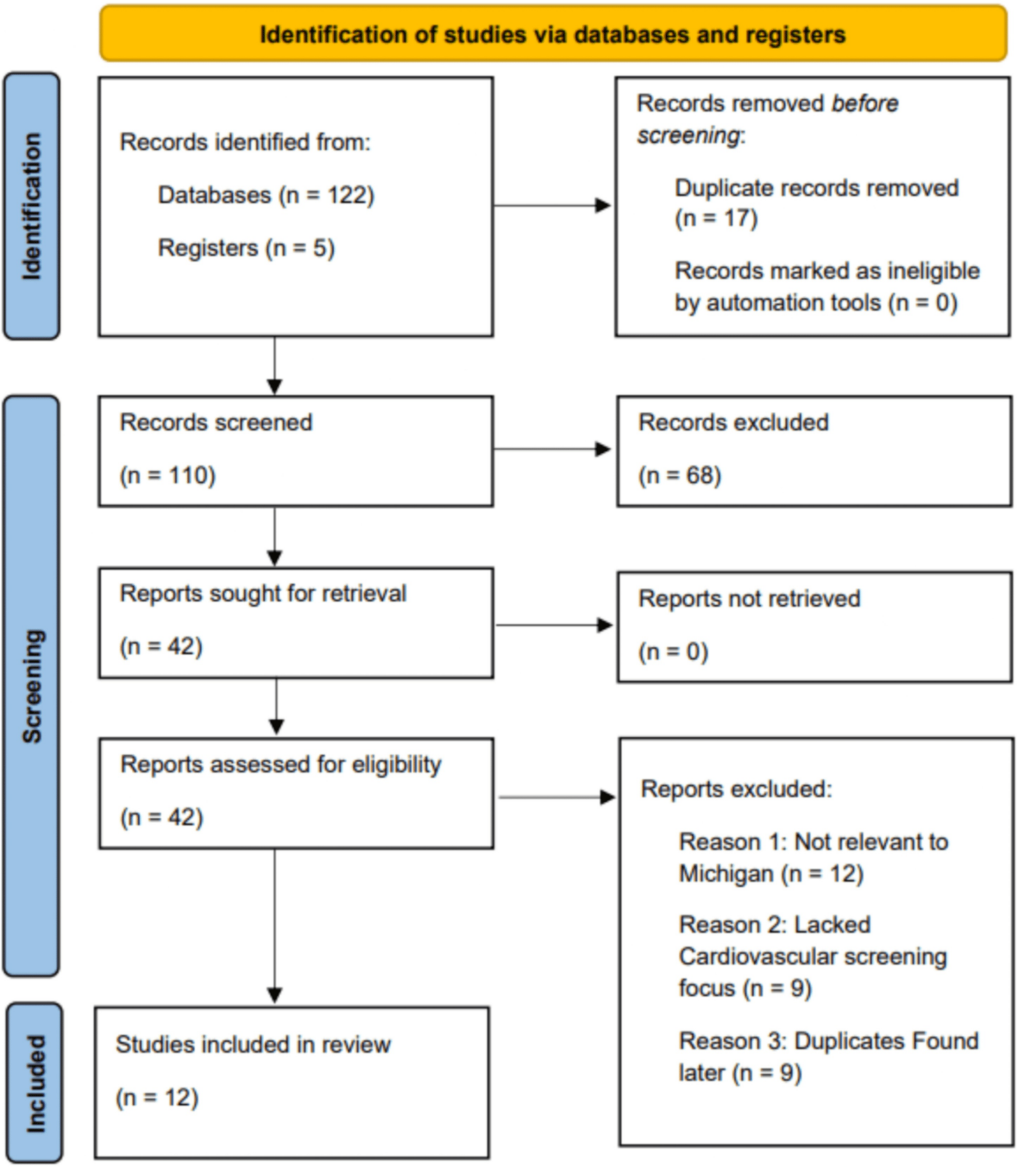
PRISMA flow diagram for the study selection process. PRISMA, Preferred Reporting Items for Systematic Reviews and Meta-Analysis

In accordance with Preferred Reporting Items for Systematic Reviews and Meta-Analysis (PRISMA) 2020 guidelines [12], we performed a comprehensive literature search. We searched PubMed, Embase, Scopus, Web of Science, and Google Scholar for studies up to October 2025, with no lower date limit. No lower date limit was applied to capture the full scope of Michigan-specific screening literature, which is limited and sporadically published. The search strategy combined free-text keywords and controlled vocabulary related to cardiovascular screening, prevention, disparities, and Michigan. Specifically, we used terms for cardiovascular health (“cardiovascular,” “hypertension,” “blood pressure,” “cholesterol,” “lipid”), screening and prevention (“screening,” “preventive,” “monitoring”), disparities (“disparities,” “inequities,” “access,” “barriers”), and the location “Michigan.” Boolean operators (AND, OR) were used to join these concepts, and search strings were customized to each database. Searches were updated in October 2025 to capture the most recent literature. We also manually screened the reference lists of relevant articles to identify additional studies. The overall selection process is summarized in the PRISMA flow diagram shown in Figure 1.

Eligibility criteria

Figure 1 shows the PRISMA flow diagram for the study selection process. Studies were included if they met the following criteria: population/setting, conducted in Michigan or using Michigan-specific data and involving human adults (>18 years); intervention/exposure, focused on preventive cardiovascular screening, specifically for hypertension or cholesterol; and study design/source, any empirical study design published in peer-reviewed journals, government or public health reports, or academic and gray literature.

Gray literature was included because Michigan-specific screening data are frequently reported through governmental and organizational sources rather than peer-reviewed journals. Inclusion was limited to reports from established public health agencies, national organizations, or reputable foundations. These sources were appraised separately and interpreted with appropriate caution.

Studies were excluded if they were conducted outside of Michigan, did not address preventive screening, or were commentaries without original data. These criteria ensured that we captured all relevant evidence on preventive hypertension or cholesterol screening disparities within Michigan from rigorous sources of data. 

Study selection

After deduplication, two reviewers independently screened the titles and abstracts of all retrieved records for potential relevance. Reviewers were not blinded to authors, institutions, or journal sources, consistent with standard systematic review practice [12]. Potential reviewer bias was mitigated through independent dual screening, standardized extraction forms, and consensus-based resolution of discrepancies. Duplicate references were removed automatically and manually verified. Records deemed potentially eligible by either reviewer were advanced to full-text review. The same two reviewers independently assessed the full text of each candidate article against the eligibility criteria. Discrepancies in either stage were resolved by discussion, with a third reviewer adjudicating if needed. We documented reasons for excluding studies at the full-text stage.

Data extraction

Data from included studies were extracted using a structured extraction table (data collection form) by two reviewers working independently. Extracted items included: study design/type; population characteristics (sample size, demographics); setting and geographic location; type of cardiovascular screening (e.g., blood pressure or cholesterol testing); disparities addressed (e.g., by race/ethnicity, socioeconomic status, rural/urban status); and key findings (including any reported screening rates or barriers identified). Any disagreements in extracted data were resolved through discussion, with consensus reached on all items. The data extraction form was developed a priori and pilot-tested on a subset of included studies to ensure consistency and clarity. The finalized data table provided the basis for our qualitative synthesis of results (Table 1).

**Table 1:** Included studies on cardiovascular screening disparities in Michigan. MI, Michigan; CVD, cardiovascular disease; BP, blood pressure; chol, cholesterol; SES, socioeconomic status; CQI, Collaborative Quality Initiative

Study (year)	Sample size	Study type	Screening type	Key findings	Barriers	Interventions
MDHHS Data and Statistics Portal (n.d.) [3]	MI counties and divisions	Dataset/surveillance	CVD mortality and risk	Geographic variation; limited screening data	Resource disparities	County-level mapping data
MiBRFSS (2019-2021) [4]	MI adults (by region)	Cross-sectional survey	Self-reported BP and cholesterol screening	Screening varies by income and region	Low income, education, rural residence	Data guides target interventions
Krefman et al. (2023) [6]	Rural MI primary-care practices	Intervention	Hypertension prevention and control	Statewide initiative for rural BP care	Infrastructure and staff shortages	“Healthy Hearts for Michigan”
Clements et al. (2020) [7]	MI Medicare older adults	Quantitative (claims)	Preventive Screening (BP, chol, diabetes)	Black and low-income individuals are less likely to be screened	Insurance gaps, rural, SES	Equity policies and provider incentives
Blue Cross Blue Shield Foundation (n.d.) [9]	Underserved communities	Foundation report	Prevention and BP control	Funded programs improved outcomes	Awareness and funding gaps	Community grants and partnerships
American Heart Association (2021) [10]	Midwest incl. MI	NGO policy report	CVD policy and prevention	Policy reforms recommended	Socio-economic barriers	Preventive-care advocacy
MDHHS (2019) [11]	MI minority groups	Gov report	Chronic disease and CVD disparities	Persistent racial gaps in outcomes	Racism, underinsurance, and access limits	Equity and social-determinant policies
Dean et al. (2024) [13]	Detroit's underserved communities	Observational (public health)	CVD risk reduction and prevention	Poverty and food insecurity, and poor screening	Inequality and education gaps	Community health outreach and lifestyle programs
Centers for Disease Control and Prevention (2023) [14]	All MI counties	Dataset/surveillance	BP and cholesterol screening	Rural counties screen less	Workforce shortages	High-risk zone targeting
Brook et al. (2022) [15]	Detroit residents	Observational (intervention evaluation)	BP and cholesterol screening	Mobile units to provide screening	Limited primary care access	Community-based intervention
Michigan Department of Health and Human Services (2020) [16]	Statewide population	Government Report	CVD screening rates	CVD leading cause; low screening for the uninsured	SES and education barriers	State campaigns and awareness programs
Austin Publishing Group (n.d.) [17]	Statewide health systems	Review/commentary	CVD interventions	Hospital collabs reduce inequities	Resource distribution issues	Michigan CQIs

Quality appraisal

Two reviewers independently assessed the methodological quality and risk of bias of each included study. To reduce selection bias, Google Scholar results were limited to the first 200 results sorted by relevance, consistent with common systematic review practice. We used established assessment tools appropriate to the study design. Observational and surveillance studies were evaluated using NIH Quality Assessment Tools, and implementation studies were appraised using relevant public health appraisal criteria. Each study was judged across relevant domains (e.g., selection bias, confounding, outcome measurement) and rated for overall quality (low, moderate, or high risk of bias). Any disagreements in quality ratings were resolved through discussion. These quality assessments were used to contextualize the strength of evidence, but did not serve as an exclusion criterion. Inter-rater reliability statistics (e.g., kappa) were not calculated, as disagreements were infrequent and resolved through discussion; risk-of-bias ratings were used to contextualize findings rather than formally weight evidence. Table 2 shows a summary of the quality appraisal ratings. 

**Table 2:** Quality assessment of included studies based on methodological rigor.

Study	Study design	Source type	Quality rating	Rationale
MDHHS (2019) [11]	Government Report	Surveillance/Secondary Data	Moderate	Reliable source with robust state-level data; lacks detailed methodology.
MiBRFSS (2019-2021) [4]	Cross-sectional survey	Surveillance Data	Moderate-High	Systematic sampling and large sample size; self-reported data introduces bias.
MDHHS (2020) [16]	Government Report	Surveillance Summary	Moderate	Authoritative and updated, but lacks methodological detail.
Clements et al. (2020) [7]	Claims Analysis	Peer-reviewed Quantitative	High	Strong design, large Medicare dataset, adjusted for confounders.
Blue Cross Blue Shield Foundation (n.d.) [9]	Foundation Report	Gray Literature	Moderate	Program evaluation: evidence of outcomes but limited statistical rigor.
Austin Publishing Group (n.d.) [17]	Commentary/Review	Gray Literature	Low	Lacks primary data or analysis; more descriptive.
American Heart Association (2021) [10]	Policy Report	NGO Report	Moderate	Nationally reputable, but lacks methodological transparency.
Brook et al. (2022) [15]	Implementation Study	Peer-reviewed	High	Describes applied methods and outcomes; moderate sample but well-designed.
MDHHS Statistics Portal [3]	Surveillance Dataset	Public Health Surveillance	Moderate	Aggregated data are useful for trends but lack granularity.
Krefman et al. (2023) [6]	Intervention Trial	Peer-reviewed Pilot	High	Field trial design, strong focus on outcomes in rural settings.
CDC (2023) [14]	Government Surveillance	National Dataset	High	Valid, comprehensive data collection methodology.
Dean et al. (2024) [13]	Observational Study	Peer-reviewed Public Health	High	Strong design, community-based, clearly addresses disparities.

All 12 studies included in this review were assessed for methodological rigor and credibility using adapted criteria based on the NIH Quality Assessment Tools and principles suited for mixed-method public health research. Of these studies, six were rated high quality. These included peer-reviewed observational studies, Medicare claims analyses, and rural intervention evaluations, all of which demonstrated robust methodologies, large or representative sample sizes, and clearly articulated outcomes [4,6,7,13,14,15]. 

Five studies were rated as moderate quality, largely consisting of government reports and foundation publications such as those from the MDHHS and Blue Cross Blue Shield of Michigan [3,9,10,11,16]. While these sources provided important population-level data, they often lacked detailed methodological transparency or peer review, limiting the ability to fully evaluate internal validity. Finally, one study, published by the Austin Publishing Group, was rated low quality due to its descriptive nature and absence of primary data or systematic analysis. Despite this, it offered contextual value on community-level screening gaps and perceptions [17].

Overall, the quality appraisal process confirmed that the review drew primarily on strong and credible sources. The inclusion of diverse evidence types also reflects the complexity of cardiovascular screening disparities in Michigan, particularly across racial, geographic, and insurance-status lines.

Results

Proportions reported in this section refer to the number of included studies addressing a given theme rather than pooled effect sizes or weighted evidence strength. Findings derived directly from Michigan-specific datasets are distinguished below from national studies cited for contextual comparison. Several included studies examined multiple disparity dimensions. These studies are discussed across relevant thematic subsections; however, findings are synthesized thematically rather than counted as independent evidence in each domain. Of the 12 included studies, 7 (58%) examined racial/ethnic disparities [4,7,9,10,11,13,16], 4 (33%) examined geographic (rural vs. urban) differences [3,4,6,11], 6 (50%) examined socioeconomic factors [7,9,10,11,13,16], and 4 (33%) addressed intervention strategies or implementation problems (Table 1) [6,9,17,15]. Themes overlapped in some studies. For example, Clements et al. found that White Medicare beneficiaries in Michigan were significantly more likely to use preventive services than racial/ethnic minorities [7]. Similarly, national analyses show that Black and Hispanic adults have lower rates of cardiovascular preventive services (e.g., blood pressure and cholesterol checks) than White adults [1,4]. Across studies, the pattern was consistent: minority populations generally had lower screening use (typically ranging from 5% to 15% points across studies). One national Medicare analysis noted that Black-White screening gaps were modestly smaller under Medicare Advantage than traditional Medicare (~2.2% point improvement in BP and cholesterol checks), but disparities persisted in all settings [3,18]. 

Racial and ethnic disparities

Most studies (8/12) reported on racial or ethnic differences in screening. All found that non-White groups were underserved relative to Whites. For instance, Black and Hispanic adults in Michigan were significantly less likely than White adults to receive routine cardiovascular screenings [4,7]. These Michigan-specific findings match larger U.S. patterns where Black adults have lower preventive care use than Whites across multiple measures, including BP and cholesterol checks. A few studies also noted disparities for other groups (e.g., Latino or Asian populations), though sample sizes were often smaller [4,7]. Notably, the magnitude of disparity varied; one analysis suggested that managed care enrollment (Medicare Advantage) slightly narrowed Black-White gaps, but they remained present. In summary, the studies consistently indicated poorer preventive screening among racial/ethnic minorities, with little evidence of the gaps converging; however, not all studies performed multivariable adjustment.

Geographic (rural vs. urban) disparities

Five studies explicitly addressed rural-urban differences. All acknowledged that rural Michigan residents bear a heavier CVD burden and have fewer health resources. In these studies, rural residence was generally associated with lower preventive screening, although few studies provided detailed urban/rural comparisons. For example, baseline data from a rural-practice hypertension program showed that only ~72% of patients had blood pressure screening documented [6]. In contrast, one statewide survey found minimal difference in cholesterol testing rates by urbanity [4]. Overall, the limited evidence suggested that screening uptake tends to be modestly lower in rural areas, reflecting Michigan’s known primary care shortages in nonmetropolitan counties [6,11]. However, the studies diverged in exact estimates, and most did not statistically adjust for confounders, making the geographic disparities less certain than the racial disparity. Most geographic comparisons were descriptive and unadjusted, limiting causal interpretation of rural-urban differences.

Socioeconomic disparities

Six studies evaluated socioeconomic gradients in screening. All reported that lower incomes or education were linked to reduced preventive care. One large national study showed that adults near poverty were roughly 55-67% less likely to have recent cholesterol or blood pressure screening than high-income adults. Similar patterns appeared in Michigan-based data: uninsured or Medicaid-enrolled patients had lower rates of annual cardiovascular risk assessments [7,11]. For example, after Michigan’s Medicaid expansion, enrollees reported increased preventive service use, highlighting prior gaps [19]. Few studies fully disentangled education vs. income, but those that did observed consistent SES-related gaps. These findings align with broader evidence that low socioeconomic status severely limits access to routine screening. A minor divergence was that one study found education effects attenuated after adjusting for insurance, suggesting insurance coverage mediates much of the SES disparity [20]. Several studies suggested that insurance coverage mediated much of the observed socioeconomic disparity, though disentangling income and education effects was limited.

Intervention strategies and implementation gaps

Four studies described intervention approaches or noted implementation gaps. Examples included community outreach and practice-based quality improvement. The Detroit mobile health unit (MHU) program was a prominent example: over one year, the MHU provided screening to >32,000 people and increasingly targeted high-vulnerability neighborhoods [20]. Authors reported that this data-driven mobile strategy “improves health equity by addressing longstanding gaps in primary care” in Detroit [8]. Another effort, the Healthy Hearts for Michigan trial, is facilitating hypertension management in rural primary care practices, although results are not yet reported [6]. Despite these interventions, none of the included studies evaluated statewide or long-term screening initiatives. Moreover, all intervention reports noted challenges such as sustaining funding, engaging patients, and tracking follow-up. In summary, the few studies on interventions suggest promising models (mobile clinics, practice facilitation) but also reveal critical gaps: lack of rigorous evaluation of screening outcomes and absence of large-scale implementation data.

Discussion

Our systematic review revealed consistent evidence of disparities in preventive cardiovascular screening in Michigan. This discussion interprets disparities in preventive screening observed in Michigan-specific data, supplemented by national evidence where Michigan-specific screening measures were limited. Racial and ethnic gaps were prominent: for example, Michigan Medicare data show that non-Hispanic Black beneficiaries with diabetes were less likely than White beneficiaries to use any preventive services [7]. National data similarly find lower odds of blood pressure or cholesterol screening among Hispanic and Asian immigrant adults compared to U.S.-born White adults [1,4]. Geographic disparities are also apparent. Rural Michigan communities bear a disproportionate burden of CVD and have fewer primary-care resources, suggesting likely gaps in screening access (as noted in Healthy Hearts for Michigan planning documents). Socioeconomic gaps were also striking; screening rates rose with higher income and insurance coverage. For instance, Michigan Behavioral Risk Factor Surveillance System (BRFSS) data (2021) show that 89.6% of insured adults had a cholesterol check in the past 5 years versus only 56.2% of uninsured [4]. Similarly, 79.5% of insured Michigan adults reported an annual doctor’s visit in the past year compared to just 34.9% of uninsured [4]. These patterns may contribute to implementation gaps between guideline recommendations and practice: even though national guidelines call for routine blood pressure, cholesterol, and diabetes screening in adults, large segments of at-risk populations in Michigan are not being reached. In sum, our review found multifaceted disparities in preventive cardiovascular care, by race/ethnicity, geography, and socioeconomic status, indicating uneven uptake of recommended screening across the state.

Interpretation in the context of U.S. research

Our Michigan-specific findings align closely with national patterns. Because Michigan-specific longitudinal screening data are limited, national studies are cited here to contextualize observed disparities rather than as direct evidence of Michigan outcomes. Broad U.S. data show that racial/ethnic minorities and socioeconomically disadvantaged groups have lower use of preventive cardiovascular services [10]. For example, a recent analysis of national Medicare survey data found that Black beneficiaries in Medicare Advantage reported more preventive CVD services than in traditional Medicare, modestly narrowing the gaps between Black and White populations; nonetheless, Black-White and Latinx-White disparities persisted [20]. Likewise, a 2024 national study using NHIS data reported that Latino and Asian immigrants had significantly lower odds of annual blood pressure screening than U.S.-born White adults. Psychosocial factors play a role nationally as well; adults reporting discrimination or life dissatisfaction had substantially reduced odds of undergoing cholesterol or glucose screening [7]. These broader findings suggest that Michigan’s disparities are not unique but part of systemic national patterns.

At the same time, some Michigan-context factors may influence these patterns. Michigan’s expansion of Medicaid under the Affordable Care Act has improved access: one IHPI study found that ~81% of Michigan Medicaid expansion enrollees had a recent primary care visit and screening rates (e.g., mammography) approaching those of the privately insured [20]. This likely helped blunt some screening gaps. However, even in Michigan, the uninsured rate in 2011 was much higher among Black and Hispanic adults (17%-20%) than among White adults (10%), and as of 2017, a residual coverage gap remained. Thus, while expanded coverage has narrowed disparities, it has not yet eliminated them.

In sum, Michigan’s patterns of lower screening in minorities, rural residents, and the uninsured reflect broader U.S. evidence. Our findings are consistent with national research demonstrating multifactorial causes, from insurance and primary care availability to discrimination and health literacy, that underlie preventive care inequities. The concordance between Michigan and national data strengthens confidence in these conclusions. Any divergence (e.g., the relatively high rate of routine checkups reported among Black Michigan residents) may reflect local initiatives (such as community outreach) and warrants further study. Overall, persistent screening gaps in Michigan have critical implications: they likely contribute to worse cardiovascular outcomes in vulnerable groups, echoing national calls for targeted equity-focused interventions.

Policy and practice implications

The documented disparities suggest several actions for Michigan stakeholders. The following implications are informed primarily by the patterns identified in the included Michigan studies, supplemented by established evidence from broader U.S. public health literature. First, expand and enhance access to health care for underserved populations. Continuing Medicaid expansion and outreach should remain a priority, since insured Michiganders had much higher screening rates than the uninsured. State programs like the Healthy Michigan Plan can be leveraged to ensure enrollment of eligible adults. Ensuring coverage for recommended preventive services without cost-sharing is critical.

Second, strengthen primary care capacity in rural and high-need areas. Michigan faces rural primary care shortages that likely impede screening. Investment in rural clinics, telemedicine, and mobile screening units (as piloted in Detroit) could help reach remote populations. The Michigan Improving Cardiovascular Health (MICH) Collaborative and Million Hearts 2027 initiatives provide frameworks to scale evidence-based CVD prevention strategies [8,21]. Health systems should monitor screening rates by county and target clinics in underserved regions for quality-improvement efforts.

Third, target disparities with culturally-tailored outreach and education. Community health workers and public health campaigns can raise awareness of the importance among Black, Hispanic, and immigrant communities. Materials should be linguistically appropriate and address trust barriers, since discrimination and low life satisfaction have been linked to lower screening uptake. Partnerships with churches, schools, and local organizations can improve reach. Health systems can train providers in cultural competency to encourage screening during visits.

Fourth, leverage data and quality metrics to drive equity. Policymakers and payers could incorporate equity measures into quality reporting (e.g., stratifying screening rates by race or ZIP code). Clinics can use Michigan’s CHRONICLE registry to flag patients overdue for blood pressure or cholesterol checks and send reminders. Embedding community need indicators in the statewide cardiovascular dashboard will help track progress. Learning from the IHPI study, Medicare Advantage and Medicaid plans in Michigan could be required to report disparities in preventive care (e.g., via a Health Equity Summary Score), so that planners can identify and address gaps [20,22].

Finally, address social determinants of health (SDOH) that impede screening. This includes improving transportation to clinics, expanding clinic hours, and integrating screening into community events. Policies to reduce systemic inequities, such as investing in education, housing, and employment in marginalized communities, will also help, although these are long-term endeavors. While not all proposed strategies have been directly evaluated in Michigan-specific screening studies, they reflect evidence-informed approaches commonly used in comparable public health settings. In the meantime, short-term supports like patient navigation and co-location of services (e.g., offering screening at food pantries or churches) may reduce SDOH barriers and boost preventive care awareness.

Strengths and limitations

This review has several strengths. We employed a broad, systematic search strategy across academic and governmental sources to capture diverse evidence on Michigan CVD screening. We synthesized data from state surveys (e.g., BRFSS), healthcare claims, and national studies to provide a comprehensive picture of disparities. Using multiple data types (self-report, claims) enhances credibility, and our inclusion of recent sources (up to 2025) ensures timeliness. Included studies varied substantially in study period (approximately 2009-2025), population characteristics, definitions of screening, and data sources, contributing to heterogeneity across findings.

However, there are important limitations to consider. The evidence based on Michigan-specific screening disparities is limited and heterogeneous. Studies varied in populations, years, and measures of “screening,” making direct comparisons difficult. Much of the data (e.g., BRFSS) are self-reported and subject to recall bias, and the cross-sectional design of most studies precludes causal inference. Our review is likely subject to publication bias, and some gray literature (local health department reports) might have been missed. We also found little data on certain groups (e.g., Native Americans, which is a Michigan-relevant group, or non-English speakers) or outcomes (such as actual reduction in CVD events). Additionally, several included sources were government or organizational surveillance reports rather than peer-reviewed primary studies. These reports are highly valuable in public health because they provide statewide, population-level data that are otherwise unavailable; however, they lack standardized methodological detail, may use inconsistent operational definitions of *screening*, and are not subject to formal peer review. Their inclusion adds contextual support to capture Michigan-specific evidence, but these characteristics may introduce measurement inconsistency and limit direct comparability with peer-reviewed studies. Finally, we focused on screening uptake rather than intermediate outcomes (like control of hypertension), so our conclusion about implementation gaps relies on process measures. These limitations suggest that while disparities are evident, the exact magnitudes may vary, and new data could change these findings.

Future directions

Several avenues for future work emerge. Research should delve deeper into the root causes and solutions for Michigan’s screening gaps. Longitudinal studies could assess how policy changes (e.g., Medicaid shifts in primary care supply) affect screening over time. Qualitative research (focus groups, interviews) with affected communities could illuminate barriers and facilitators in screening. County-level and clinic-level surveillance of screening rates by race/insurance would help target resources more precisely. 

Intervention studies are needed to test promising models. For example, Michigan could pilot and evaluate MHUs in rural counties or *health fairs* offering free BP/cholesterol checks in underserved Detroit neighborhoods. Implementation science approaches can identify which strategies (e.g., telehealth consults, provider prompts, and patient incentives) most effectively increase screening among disadvantaged groups. Partnerships between academic centers, the state health department, and community organizations would facilitate these studies.

On the policy front, Michigan should continue to monitor the impact of health reforms. For instance, tracking whether further narrowing of uninsured rates correlates with rising screening. Evaluating the Michigan Million Hearts and MICH Collaborative initiatives will be crucial: these programs explicitly aim to reduce CVD disparities, and their outcomes should inform future efforts. Policymakers might also explore system-level changes, such as reimbursing community health workers for screening outreach or requiring equity audits in health plans.

In the long term, research and policy should move beyond screening to comprehensive risk management. That includes ensuring follow-up of abnormal results, improving medication access, and addressing lifestyle risk factors. Finally, aligning Michigan’s efforts with national goals (e.g., Healthy People 2030/2040 or Million Hearts 2027) will help maintain momentum [13,23]. If Michigan can translate these findings into concerted actions, the state may significantly improve cardiovascular health equity in the years ahead.

## Conclusions

This review synthesizes evidence on disparities in screening utilization rather than directly measuring downstream cardiovascular outcomes. Minority, low-income, and rural populations were consistently less likely to receive recommended screenings than their White, wealthier, or urban counterparts. For example, national data are cited for contextual comparison and show that Black Medicare enrollees report lower rates of cardiovascular preventive services than White enrollees, while Michigan-specific evidence indicates that rural regions face higher CVD burden alongside primary care shortages. These findings align with broader evidence of SES- and race-based disparities: low-income adults are significantly less likely to be screened for hypertension and hyperlipidemia than high-income adults. In short, this review highlights persistent racial, socioeconomic, and geographic inequities in preventive CVD screening in Michigan, consistent with broader national patterns.

Taken together, these disparities highlight ongoing gaps in preventive cardiovascular screening. Michigan's public health agencies have begun equity-focused initiatives; for example, the MICH Collaborative has promoted multi-sector strategies aimed at improving cardiovascular health equity through enhanced detection and management of risk factors, and state heart disease and stroke prevention programs have reported targeted outreach in high-burden Detroit and Flint neighborhoods. However, the evidence-practice gap remains wide: few rigorously evaluated screening interventions have been implemented statewide. Policymakers, health systems, and researchers may benefit from acting on this evidence by scaling community-based screening programs, improving access in underserved areas, and embedding equity metrics in quality monitoring. Such approaches may help improve equity in access to preventive cardiovascular screening and support earlier identification of cardiovascular risk among underserved populations.

## References

[1] Centers for Disease Control and Prevention (CDC) (2024). Heart Disease Facts. https://www.cdc.gov/heart-disease/data-research/facts-stats/index.html.

[2] U.S. Department of Health and Human Services (2020). Healthy People 2030: Cardiovascular Disease Objectives. Prevention and Health Promotion.

[3] Michigan Department of Health and Human Services (MDHHS) (2022). Cardiovascular Disease Mortality in Michigan. Cardiovascular Disease in Michigan.

[4] Michigan Behavioral Risk Factor Surveillance System (MiBRFSS) (2019). 2019-2021 Michigan Behavioral Risk Factor Survey Data Tables. https://www.michigan.gov/en/mdhhs/keep-mi-healthy/communicablediseases/epidemiology/chronicepi/bfrs/anntables/michigan-brfs-annual-tables.

[5] Couch C, Mascarenhas R, Stierman B, Fryar C, Zablotsky B Prevalence of cardiovascular disease risk factors in adults. NCHS Data Brief.

[6] Krefman AE, Ciolino JD, Kan AK (2023). Rationale and design for Healthy Hearts for Michigan (HH4M): a pragmatic single-arm hybrid effectiveness-implementation study. Contemp Clin Trials Commun.

[7] Clements JM, West BT, Harissa B, Hayden N, Khan MM, Palepu R (2020). Race disparities in the use of prevention, screening, and monitoring services in Michigan Medicare beneficiaries with type 2 diabetes and combinations of multiple chronic conditions. Clin Diabetes.

[8] Levy P, McGlynn E, Hill AB (2021). From pandemic response to portable population health: a formative evaluation of the Detroit mobile health unit program. PLoS One.

[9] Blue Cross Blue Shield of Michigan Foundation (2022). Closing the Gap: Cardiovascular Screening Access in Michigan’s Underserved Communities. Detroit, MI: BCBSM Foundation.

[10] American Heart Association (2021). Health Equity in Heart Disease and Stroke: Midwest Regional Report. https://www.heart.org/-/media/Annual-Report/2021-2022-Annual-Report-Files/2021_2022_American_Heart_Association_Annual_Report.pdf.

[11] Michigan Department of Health and Human Services (MDHHS) (2019). Health Risk Behaviors Within The State of Michigan. Heart Disease and Stroke in Michigan.

[12] Page MJ, McKenzie JE, Bossuyt PM (2021). The PRISMA 2020 statement: an updated guideline for reporting systematic reviews. British Med J.

[13] Dean Caress A, Rebecca M, Maidah R, Swathi R (2024). Reducing Detroit’s heart disease risk: the role of social determinants of health and health behaviors. Michigan J Pub Health.

[14] Centers for Disease Control and Prevention (CDC) (2024). Interactive Atlas of Heart Disease and Stroke: Michigan County Data. https://www.cdc.gov/heart-disease-and-stroke-data/index.html.

[15] Brook RD, Dawood K, Foster B (2022). Utilizing mobile health units for mass hypertension screening in socially vulnerable communities across Detroit. Hypertension.

[16] Michigan Department of Health and Human Services (MDHHS) (2020). Cardiovascular Health Disparities by Geography and Income in Michigan. Cardiovascular Health Disparities by Geography.

[17] Austin Publishing Group (2025). How Michigan Collaboratives Reduce Disparities. Austin J Cardiovasc Dis Atherosclerosis.

[18] National Institutes of Health (NIH) (2021). Quality Assessment Tools for Observational Cohort and Cross-Sectional Studies. https://www.nhlbi.nih.gov/health-topics/study-quality-assessment-tools.

[19] Patel MR, Tipirneni R, Kieffer EC (2020). Examination of changes in health status among Michigan Medicaid expansion enrollees from 2016 to 2017. JAMA Netw Open.

[20] Tipirneni R, Stefanescu AR, Ruggiero DA, Hames AG, Ayanian JZ, Roberts ET (2025). Racial and ethnic disparities in preventive and chronic disease care in Medicare Advantage vs. traditional Medicare. J Gen Intern Med.

[21] Ritchey MD, Wall HK, Owens PL, Wright JS (2018). Vital signs: state-level variation in nonfatal and fatal cardiovascular events targeted for prevention by Million Hearts 2022. MMWR Morb Mortal Wkly Rep.

[22] Michigan Improving Cardiovascular Health (MICH) (2025). Michigan Improving Cardiovascular Health (MICH) Learning Collaborative. https://www.michigan.gov/mdhhs/keep-mi-healthy/chronicdiseases/cardiovascular/mich-learning-collaborative.

[23] Buchmueller TC, Cliff BQ, Levy H (2020). The benefits of Medicaid expansion. JAMA Health Forum.

